# Patientensteuerung und Eigenverantwortung als Teil einer Reform der gesetzlichen Krankenversicherung: Ansätze für Deutschland

**DOI:** 10.1007/s00103-026-04292-w

**Published:** 2026-09-08

**Authors:** Andreas Beivers, Annette Höhmann

**Affiliations:** 1 Fachbereich Wirtschaft & Medien, Hochschule Fresenius, Infanteriestraße 11a, 80797 München, Deutschland; 2 Fachbereich Wirtschaft & Medien, Hochschule Fresenius, Im Mediapark 4d, 50670 Köln, Deutschland

**Keywords:** Primärarztsystem, Patientensteuerung, Eigenverantwortung, Gesundheitsökonomie, Anreizsysteme, Primary care system, Patient management, Personal responsibility, Health economics, Incentive systems

## Abstract

Vor dem Hintergrund der prekären finanziellen Lage der Gesetzlichen Krankenversicherung (GKV) wird die Einführung eines Primärarztsystems, bei dem Patienten zunächst eine hausärztliche Praxis aufsuchen und von dort bei Bedarf an einen Facharzt überwiesen werden, in Deutschland derzeit intensiver diskutiert. Ziel ist es, die Versorgung stärker zu koordinieren und die Effizienz im Gesundheitswesen zu erhöhen.

Dieser Diskussionsbeitrag zeigt auf, inwiefern ein Primärarztsystem ein wesentlicher Baustein einer grundlegenden Transformation des Gesundheitswesens sein kann und welche systemischen Voraussetzungen hierfür notwendig sind. Neben einem Blick in das europäische Ausland werden auch Erfahrungen aus Deutschland erörtert und Empfehlungen abgegeben. Dabei wird eine Neudefinition der Eigenverantwortung ebenso diskutiert wie die Möglichkeiten von Anreizsystemen für eine nachhaltige Etablierung eines solchen Systems. Bonusprogramme nach dem Vorbild erfolgreicher Loyalitätsmodelle könnten die Chance bieten, Patientensteuerung wirksam, akzeptiert und sozial ausgewogen zu gestalten.

Patientensteuerung ist keine Frage von Disziplin, sondern von Systemdesign. Eine Transformation der GKV, die Patientensteuerung und eine zeitgemäße Definition von Eigenverantwortung integriert, stärkt nicht nur die Wirtschaftlichkeit, sondern auch die Solidarität. Patientensteuerung und Eigenverantwortung sind keine moralischen Appelle, sondern ökonomische Notwendigkeiten. Ein solidarisches System bleibt nur dann stabil, wenn es effizient organisiert ist.

## Abstract (en)

Against the backdrop of the precarious financial situation of Germany’s statutory health insurance system (GKV), the introduction of a primary care physician system, in which patients first consult a general practitioner and are referred to a specialist if necessary, is currently the subject of intense debate. The aim is better coordination of care and an overall increase of efficiency in the healthcare system.

This discussion paper examines the extent to which a primary care physician system can serve as a key component of a fundamental transformation of the healthcare system and identifies the systemic prerequisites for this. In addition to examining other European countries, the paper also discusses experiences from Germany and offers recommendations. It explores a redefinition of personal responsibility as well as the potential of incentive systems for the sustainable establishment of such a system. Bonus programs modeled after successful loyalty schemes could offer an opportunity to design patient management in a way that is effective, accepted, and socially balanced.

Patient management is not a matter of discipline, but of system design. A transformation of the statutory health insurance system that integrates patient management and a modern definition of personal responsibility strengthens not only economic efficiency but also solidarity. Patient management and personal responsibility are not moral appeals, but economic necessities. A solidarity-based system remains stable only if it is efficiently organized.

## Einleitung

Die seit Jahrzehnten bekannte Finanzierungsnotlage der Gesetzlichen Krankenversicherung verschärft sich aktuell massiv. Vorschläge für Gegenmaßnahmen werden der Lage jedoch nur selten gerecht und erschöpfen sich zumeist in Überlegungen zur Erhöhung von Beiträgen oder der Streichung von Leistungen.

Vor diesem Hintergrund wurde der erste Bericht der Finanzkommission Gesundheit im März 2026 mit Spannung erwartet. Dieser Bericht enthält primär Empfehlungen zur Stabilisierung des GKV-Beitragssatzes ab 2027. Eine detailliertere Ausarbeitung der Idee einer Primärversorgung, wie es die Kommission nennt, ist erst in einem zweiten Bericht im Dezember 2026 zu erwarten, aber schon jetzt hat sich die Kommission diesbezüglich deutlich geäußert und empfiehlt die Etablierung einer bedarfsgerechten Patientensteuerung [1]. Sie sieht in der Einführung eines Primärversorgungssystems, bei dem Patienten zunächst eine hausärztliche Praxis oder ein Primärversorgungszentrum aufsuchen und von dort bei Bedarf an eine fachärztliche Praxis überwiesen werden, das Potenzial, vorhandene Ressourcen effizienter zu nutzen, die Eigenverantwortung von Patienten zu stärken und schlussendlich auch die Versorgungsqualität zu steigern. Damit kann ein Primärversorgungssystem ein wesentlicher Baustein einer notwendigen Transformation des Gesundheitswesens sein.

In diesem Diskussionsbeitrag wird erörtert, inwiefern Patientensteuerung zur besseren Ressourcenallokation im Gesundheitswesen führen kann und welcher Voraussetzungen es dafür bedarf. Ein internationaler Vergleich zeigt, dass in Ländern, in denen Patientensteuerung fester Teil des Gesundheitswesens ist, die Versorgungsqualität häufig besser ist als hierzulande. Wesentlich für den Erfolg eines Primärarztsystems ist die Akzeptanz durch die Patienten. Geeignete Anreize sollten dazu beitragen, dass sie eine koordinierte Patientensteuerung als ebenso selbstverständlich wahrnehmen wie beispielsweise die Kunden einer Airline die Teilnahme an einem Vielfliegerprogramm. Der Beitrag schließt daher mit einem Vergleich, welche Aspekte solcher Programme bei der Einführung eines Primärarztsystems wertvolle Inspiration bieten können.

## Die Ausgangslage

Seit den 1980er-Jahren gibt es immer wieder Debatten zur finanziellen Tragfähigkeit der GKV. Seit der Schaffung des Gesundheitsfonds und des Basistarifs der Privaten Krankenversicherung (PKV) im Jahr 2009 gab es allerdings keine grundlegenden Reformbemühungen mehr, obwohl die demografische Entwicklung seit Langem auf eine Verschärfung der Finanzierungsprobleme hindeutete: Immer mehr Angehörige der geburtenstarken Babyboomer-Jahrgänge erreichen das Rentenalter, wodurch sich die strukturellen Probleme der GKV weiter verschärfen werden [2].

Was regelmäßig diskutiert wird, sind Einzelmaßnahmen zur Minderung der Finanzierungsprobleme. Diese sind jedoch häufig mit unerwünschten Folgeproblemen verbunden, seien es steigende Beitragssätze (und damit steigende Lohnnebenkosten), eine Erhöhung der Zuzahlungen für Medikamente (und damit eine stärkere finanzielle Belastung von Patienten) oder die Ausgliederung wesentlicher Präventivleistungen wie der zahnmedizinischen Versorgung (und damit eine Verlagerung und vermutlich Erhöhung von Kosten weg von der Prävention hin zum stationären Sektor; [3]). Zudem greifen solche Vorschläge zu kurz, denn es bedarf keiner singulären Reform von Einnahmen und Ausgaben, sondern einer weitreichenden Transformation in allen Bereichen der Versorgung.

Diese Transformation betrifft nicht nur die medizinische Leistungserbringung selbst, sondern auch Governance‑, Finanzierungs‑, Organisationsstrukturen sowie die technologische Infrastruktur. Es würden dabei nicht nur Einnahmen und Ausgaben optimiert, sondern anhand passender Anreize Nachfrage- und Angebotsverhalten substanziell verändert. Auf der Nachfrageseite gäbe es neue Formen und Wahloptionen in Versicherungsverträgen, die auch Steuerungsansätze enthalten, und auf der Leistungserbringungsseite eine Neujustierung von Vergütungsanreizen. Ein Beispiel für solche Maßnahmen sind regionale Gesundheitsbudgets, die Anreize in Richtung Prävention und Ergebnisqualität (subjektiv wie objektiv) vorsehen [4].

Eine solche Transformation, in deren Rahmen strukturelle Reformen, Digitalisierung, Ambulantisierung, Eigenverantwortung und innovationsfreundliche Rahmenbedingungen gleichzeitig vorangetrieben werden [5], wurde lange verschleppt und muss nun so zügig umgesetzt werden, dass sie zeitnah Effekte hervorbringt und zugleich gesellschaftliche Akzeptanz erfährt. Für dieses komplexe Unterfangen scheint die „Ruckrede“ des damaligen Bundespräsidenten Roman Herzog im Jahr 1997 aktueller denn je: „Durch Deutschland muss ein Ruck gehen. Wir müssen Abschied nehmen von liebgewordenen Besitzständen … Alle sind angesprochen, alle müssen Opfer bringen, alle müssen mitmachen“ [6].

Die Finanzkommission Gesundheit empfiehlt, eine bedarfsgerechte Patientensteuerung vorrangig über die Ausgestaltung der medizinischen Versorgung zu organisieren, etwa durch die Einführung von Patientenpfaden. Auch wenn sie grundlegende Strukturreformen zwar erst in einem zweiten Bericht im Dezember 2026 vorlegen möchte, ist der Ansatz, Patientensteuerung durch ein Primärarztsystem zu etablieren, schon jetzt ein Vorschlag, der wesentlicher Treiber der notwendigen Transformation sein kann [7].

## Steuerung und Eigenverantwortung neu denken

Das deutsche Gesundheitssystem ist stark angebotsgetrieben und sektoral organisiert. Versicherte haben – unabhängig von medizinischer Dringlichkeit – weitgehend freien Zugang zu Fachärzten und Notaufnahmen. Dieses System war lange Ausdruck eines hohen Freiheitsversprechens. In Zeiten knapper Ressourcen wird es jedoch zu einem Effizienzproblem. Überfüllte Notaufnahmen, lange Wartezeiten bei Fachärzten und Doppeluntersuchungen sind Effekte hiervon, die in der öffentlichen Debatte breit diskutiert werden. Sie sind nicht nur die Folgen einer scheinbar mangelnden Disziplin der Versicherten, sondern auch Ausdruck fehlender Strukturierung. Die GKV kennt bislang kaum systematische Wege, um die Inanspruchnahme von Leistungen sinnvoll zu lenken. Patientensteuerung ist daher kein Eingriff in die Versorgungsfreiheit, sondern eine notwendige Modernisierung der Systemarchitektur.

In einem derart modernisierten System kann Patientensteuerung kombiniert werden mit möglichen Anreizen wie Eigenbeteiligung oder Versicherungsverträgen mit und ohne Arztbindung. Ein Blick in die Verhaltensökonomie zeigt zudem, dass geeignete Anreize zur Realisierung bedarfsgerechter Steuerung nicht nur monetärer Natur sein müssen. Ein Beispiel für nichtmonetäre Anreize, das bereits im Gesundheitswesen Anwendung findet, ist Nudging (englisch für „sanftes Anstupsen“), also die gezielte Gestaltung von Entscheidungssituationen, um gesundheitsförderliche oder anderweitig erwünschte Entscheidungen wahrscheinlicher zu machen, ohne die Wahlfreiheit einzuschränken. Dies kann im Bereich der Prävention beispielsweise die Art und Weise sein, wie in Kantinen gesündere Gerichte präsentiert werden oder welchen Umfang einzelne Portionen gesundheitsschädlicher Nahrungsmittel wie Süßigkeiten oder Softdrinks haben. Auch die seit Jahren üblichen Abschreckungsbilder auf Zigarettenpackungen folgen dem Prinzip des Nudgings [8].

Der in einem Primärarztsystem integrierte Steuerungsgedanke kann zudem nicht nur der Ressourcenlenkung dienen, sondern auch die (Eigen‑)Verantwortung der Versicherten stärken. Der Begriff der Eigenverantwortung wird in gesundheitspolitischen Debatten bisher recht uneinheitlich verwendet. Er dient als Sammelbezeichnung für sehr unterschiedliche Forderungen – von erweiterten Wahlmöglichkeiten beim Leistungsumfang oder bei der Versorgungsform über höhere Eigenbeteiligungen bis hin zu einem verantwortungsvollen Umgang mit der eigenen Gesundheit. Gerade diese begriffliche Unschärfe macht deutlich, dass Eigenverantwortung derzeit vor allem als politisches Schlagwort fungiert, dem meist kein systematisch entwickeltes und klar konturiertes Konzept zugrunde liegt [9]. Die Folge ist, dass zentrale Dimensionen von Eigenverantwortung leicht aus dem Blick geraten und der Begriff häufig als reine Kostenbeteiligung verstanden wird, etwa in Form von Zuzahlungen oder Selbstbehalten. Dies passt zum typischen Muster wirtschaftspolitischer Diskussionen – grundsätzlich rückt Eigenverantwortung immer dann in den Fokus gesellschaftlicher Debatten, wenn die wirtschaftliche Lage als eher schlecht eingeschätzt wird [10]. Eine Beschränkung der Eigenverantwortung auf eine finanzielle Beteiligung an GKV-Leistungen greift jedoch zu kurz. Eine moderne Eigenverantwortung bedeutet informierte Mitentscheidung auf Basis transparenter Informationen, Nutzung geeigneter Versorgungswege, Teilnahme an Präventions- und Behandlungsprogrammen sowie einen bewussten Umgang mit Ressourcen [11].

Um Eigenverantwortung von Patienten im Hinblick auf ihr Nachfrageverhalten erwarten zu können, muss das Gesundheitssystem verständlich und navigierbar sein, sodass sich Patienten eigenständig orientieren können [10]. Hierfür sind 3 Voraussetzungen wesentlich, auf die im Folgenden näher eingegangen wird: eine verbindliche und zugleich nutzerfreundliche Patientensteuerung, geeignete Anreizsysteme sowie Transparenz über Kosten und Nutzen von Gesundheitsleistungen.

### Nutzerfreundliche Patientensteuerung.

Zentraler Bestandteil einer GKV-Transformation sollte eine strukturierte Patientensteuerung als Regelleistung sein. Dazu gehört eine standardisierte Ersteinschätzung, insbesondere vor ungeplanten Facharzt- oder Notfallkontakten. Eine qualifizierte Triage – digital oder telefonisch – sollte als Standardzugang etabliert werden. Ziel ist nicht Abschreckung, sondern ihre schnelle Zuweisung in das jeweils geeignete Versorgungslevel [12]. Dies wird bereits mit der sich im Gesetzgebungsverfahren befindlichen Notfall- und Rettungsreform in die Wege geleitet.

Zentral für die Nutzerfreundlichkeit eines solchen Systems ist die adäquate Einschätzung im Elektivfall. Triagierungskonzepte aus der Notfallmedizin können methodisch nicht auf Ersteinschätzungen, ob beispielsweise ein Facharzttermin vonnöten ist, übertragen werden. Dies erfolgt im Status quo primär über Hausärzte und es müssen klare und standardisierte Kriterien erarbeitet werden, wie diese die Ersteinschätzung vornehmen können.

### Geeignete Anreizsysteme.

Eine stärkere (hausärztliche) Koordination sollte mit konkreten Vorteilen verbunden sein, beispielsweise einer niederschwelligen digitalen Kommunikation oder einer Versorgung durch multiprofessionelle Teams. Im besten Fall sind die Steuerungssysteme in ihrem Gesamtkonzept so attraktiv, dass Versicherte sie aufgrund der spürbaren Serviceverbesserung einer direkten Inanspruchnahme fachärztlicher Leistungen vorziehen [13].

Zudem zeigen internationale Erfahrungen und verhaltensökonomische Erkenntnisse deutlich, dass positive Anreize häufig wirksamer sind als Sanktionen. Die GKV sollte deshalb Bonus- und Anreizmodelle systematisch ausbauen, damit eine koordinierte Versorgung von den Patienten als attraktivste Option wahrgenommen wird.

### Transparenz über Kosten und Nutzen von Leistungen.

Versicherte müssen strukturell in die Lage versetzt werden, eigenverantwortliche Entscheidungen zu treffen. Hierfür benötigen sie transparente Informationen über Versorgungsangebote, empfohlene Pfade und zu erwartende Wartezeiten. Daher sollte eine bundesweit interoperable Versorgungsnavigation Teil der Telematikinfrastruktur werden.

Hinzu kommt, dass viele Versicherte nicht wissen, welche Kosten einzelne Leistungen verursachen oder welche Alternativen bestehen. Im Rahmen einer GKV-Transformation sollten daher patientenverständliche Kosteninformationen bereitgestellt, Feedback zur eigenen Inanspruchnahme ermöglicht und Versorgungsentscheidungen transparenter gemacht werden, und zwar nicht als Instrument der Rationierung, sondern als wesentliche Grundlage informierter Entscheidungen.

## Gesundheitsökonomischer Blick auf ein Primärarztsystem

Eines der größten Nadelöhre in der deutschen Versorgungslandschaft stellt die hausärztliche Versorgung dar. Der demografische Wandel verschärft dieses Problem; Stand Dezember 2024 waren knapp 45 % der niedergelassenen Allgemeinmediziner in Deutschland 60 Jahre oder älter [14]. Mittelfristig droht daher insbesondere in ländlichen Regionen eine Unterversorgung, da viele Hausärzte keinen Nachfolger finden [15].

Vor diesem Hintergrund greift es zu kurz, sich bei der Einführung eines Primärarztsystems einzig auf die Rolle der Hausärzte im Status quo zu fokussieren. Es braucht ein ganzes Bündel an Maßnahmen, damit Patientensteuerung über alle Versorgungsbereiche hinweg funktioniert. Dies umfasst auch die Notfallversorgung, ebenso wie ein ganzheitliches Case-Management über den Versorgungspfad hinweg [4].

Wesentlich für den Erfolg eines Primärarztsystems ist, dass Hausärzte einen deutlich kleineren administrativen Aufwand haben als aktuell. Dies kann durch die Einbeziehung weiterer Akteursgruppen erreicht werden. Insbesondere in Regionen mit einer gefährdeten hausärztlichen Versorgung können die im Krankenhausversorgungsverbesserungsgesetz (KHVVG) und im Krankenhausreformanpassungsgesetz (KHAG) vorgesehenen sektorenübergreifenden Versorger eine wichtige Rolle bei der Patientensteuerung übernehmen. Generell kann auch die ärztliche Kompetenz der stationären Versorgung mit einbezogen werden. Darüber hinaus stellen neue Berufsbilder, wie bspw. Physician Assistants, oder qualifiziertes pflegerisches Personal mögliche Ressourcen dar, die eine Steuerungsfunktion übernehmen können. Schlussendlich können telefonische und KI-gestützte digitale Angebote Kapazitätsengpässe abfedern [16].

Als klarer monetärer Anreiz kann Patienten, die sich steuern lassen, ein verbesserter Tarif angeboten werden. Bei der Festlegung der Beitragsdifferenz zwischen Direktzugang und Steuerung muss allerdings vermieden werden, dass Versicherte mit hohen Beiträgen, die eine wichtige Säule der Finanzierung der GKV sind, für den Direktzugang deutlich stärker belastet werden als Versicherte mit niedrigem oder keinem Einkommen. Dies wäre sowohl aus verteilungspolitischer Sicht als auch in Hinblick auf die Gerechtigkeit des Systems problematisch [17].

Wie oben bereits illustriert, sollte die Patientensteuerung eine so hohe Prozess- und Behandlungsqualität aufweisen und durch passende Anreize so attraktiv gestaltet werden, dass sie als Qualitätsmerkmal der Versorgung verstanden wird und damit – ganz im Sinne des Nudgings – als naheliegende Wahl. Dazu gehört auch, durch entsprechende Steuerungsmechanismen zu verhindern, dass (Facharzt‑)Termine von Patienten nicht wahrgenommen werden und so bei gleichzeitig immer längeren Wartezeiten ungenutzte Behandlungskapazitäten entstehen [16].

Dasselbe gilt für mögliche negative Effekte, die aus einem Primärarztsystem entstehen können und teilweise in anderen Ländern beobachtbar sind. Dazu gehört ein generell beschränkter Zugang zu medizinischen Leistungen, die starke Abhängigkeit vom Hausarzt und dessen Qualifikation, der bürokratische Aufwand der Steuerung und häufig lange Wartezeiten. Es zeigt sich außerdem, dass ein Primärarztsystem an der Diskrepanz der Versorgung zwischen Ballungs- und ländlichen Räumen vermutlich wenig ändern würde [18].

Wie in diesem Absatz deutlich wurde, ist Patientensteuerung nicht allein Aufgabe der Hausärzte, sondern eine Systemleistung, die entsprechend organisiert werden muss:

- Der Gesetzgeber muss den rechtlichen Rahmen für Anreizmodelle erweitern.
- Der Gemeinsame Bundesausschuss sollte verbindliche Standards für Navigation und Ersteinschätzung definieren.
- Krankenkassen sollten stärker in die aktive Steuerung einbezogen werden.
- Multiprofessionelle Teams müssen strukturell und finanziell gestärkt werden.

## Primärarztsysteme in internationaler Perspektive

Primärarztsysteme, bei denen Hausärzte als zentrale Gatekeeper fungieren, sind international eher die Regel als die Ausnahme [19, 20]. In Europa sind solche Systeme in einer ganzen Reihe von Ländern vorzufinden, insbesondere in staatlichen Gesundheitssystemen [21].

Die Vorteile dieser Modelle sind gut dokumentiert: Sie fördern die Kontinuität der Versorgung, verbessern die Koordination zwischen Fachärzten und stationären Einrichtungen, verhindern Doppeluntersuchungen, stärken die Prävention und tragen zu einer Kostenkontrolle im Gesundheitssystem bei [21, 22]. In OECD-Ländern mit Primärarztsystem treten zudem deutlich weniger vermeidbare Krankenhauseinweisungen aufgrund chronischer Atemwegserkrankungen und Herzinsuffizienz auf als in Deutschland [23].

Zwei konkrete Beispiele: In den Niederlanden sind nahezu alle Bürger bei einem Hausarzt registriert, der über alle Überweisungen entscheidet. In Dänemark wird die primärärztliche Praxis zusätzlich als Datenhub genutzt, um epidemiologische Informationen zu sammeln und Versorgungsprozesse effizient zu steuern. Dies verdeutlicht, dass ein Primärarzt nicht nur aus gesundheitspolitischer, sondern auch aus ökonomischer Perspektive einen erheblichen Nutzen bringen kann [21, 22, 24].

In europäischen Ländern mit etabliertem Primärarztsystem sind oft hohe Einschreibequoten von über 90 % zu beobachten. In den genannten Nachbarländern ist die Registrierung teils verpflichtend, teils faktisch obligatorisch, um Nachteile wie höhere Zuzahlungen zu vermeiden. Zusätzlich sind Anreize wie Prämienersparnisse von bis zu 20 % verbreitet. In Deutschland hingegen sind die Anreize zur Teilnahme an der aktuellen freiwilligen „Hausarztzentrierten Versorgung“ (HZV) deutlich schwächer und die Einschreibequote mit ca. 12 % aller Versicherten entsprechend niedriger [19–21].

Besonders hervorzuheben ist, dass in den zum Vergleich herangezogenen Ländern interprofessionelle Teams der Standard sind, in denen Hausärzte, Pflegefachpersonen und weitere Gesundheitsberufe eng zusammenarbeiten. In Deutschland wird die ambulante Versorgung dagegen nach wie vorüberwiegend durch Einzelpraxen geprägt. Zudem spielen akademisch qualifizierte Pflegefachpersonen wie auch Physician Assistants bisher kaum eine Rolle in der Primärversorgung.

Wesentliche Unterschiede zeigen sich auch bei der digitalen Unterstützung der Patientensteuerung und -verwaltung. In vielen Ländern gehören elektronische Patientenakten und digitale Kommunikationswege zwischen Primärversorgung, Fachärzten und Krankenhäusern zum Versorgungsalltag. In Deutschland steht die elektronische Patientenakte dagegen erst am Beginn der flächendeckenden Einführung [16].

Der Vollständigkeit halber seien aber auch Kritikpunkte an der Patientensteuerung benannt, die beim Blick über die Landesgrenzen sichtbar werden Dazu zählen die mögliche Einschränkung der freien Arztwahl und ein Anstieg der Arbeitslast der Primärärzte, wenn sie in einem entsprechenden System die Rolle des Gatekeepers übernehmen [21, 22, 24].

Tab. 1 gibt einen Überblick über die zentralen Aspekte von Primärarztsystemen in europäischen Nachbarländern.

**Tab. 1:** Vergleich zentraler Merkmale von Primärarztsystemen in Deutschland und ausgewählten europäischen Ländern [21]

Aspekt	Deutschland (HZV/geplantes Primärarztsystem)	Europäische Vergleichsländer
**Teilnahme/Registrierung**	Freiwillig *(HZV, ~12% der Bevölkerung eingeschrieben)*	Meist verpflichtend oder faktisch obligatorisch; Einschreibequoten oft > 90 %
**Anreizmechanismen**	Kleine Boni *(z.B. Zuzahlungsbefreiungen, Gesundheits-Check-ups)*	Oft deutlich stärkere Anreize: z. B. bis zu 20 % Prämienersparnis *(Schweiz), *höhere Zuzahlungen bei Nichtteilnahme *(Niederlande, Frankreich)*
**Struktur der Versorgung**	Hausärztliche Einzelpraxis dominiert	Häufig sind interprofessionelle Teams in Gesundheitszentren tätig *(Hausärzte, Pflegekräfte, Psychologen, Physiotherapeuten etc.)*
**Rolle von Pflegefachkräften**	Akademisch qualifizierte Pflegekräfte kaum eingebunden	Advanced Practice Nurses (APN) übernehmen in vielen Ländern *(z.B. Vereinigtes Königreich, Finnland, Norwegen) *eigenständige Aufgaben, inkl. Verschreibung bestimmter Medikamente
**Zugang zu Fachärzten**	Überweisung durch Hausarzt in HZV erforderlich, sonst freie Arztwahl	In fast allen Ländern ist Überweisung zwingend nötig; Ausnahmen mit Eigenanteilen oder geringeren Erstattungen *(z.B. Frankreich, Schweiz)*
**Digitalisierung**	Elektronische Patientenakte im Aufbau, bislang geringe Verbreitung	In vielen Ländern automatischer digitaler Informationsaustausch zwischen Versorgern *(z.B. Finnland, Italien, Schweiz, Dänemark)*
**Politische Entwicklung**	Neues verbindliches Primärarztsystem seit 2025 politisch gefordert, Ausgestaltung offen	Meist bereits seit Jahren etabliert, kontinuierlich weiterentwickelt

## Bonusprogramme für Patienten

Wie bereits erläutert, muss ein nachhaltiges Patientensteuerungsmodell systemisch gedacht werden. Für die Einführung sind Erkenntnisse der Verhaltensökonomie wesentlich, denn Patienten handeln selten rein rational [25]. Zeitdruck, Unsicherheit, Bequemlichkeit und Angst prägen im Krankheitsfall Entscheidungen stärker als formale Versorgungslogiken. Wer Beschwerden hat, sucht den direkten, sicheren Weg und dieser führt häufig in die Notaufnahme oder direkt zu einem Facharzt. Ein Primärarztsystem, das den Anschein erweckt, diesen Weg zu verschließen, wird Akzeptanzprobleme erzeugen und Ausweichverhalten fördern.

Der oben illustrierte Blick ins Ausland zeigt, dass Steuerung dann am besten funktioniert, wenn sie für Patienten spürbar vorteilhaft ist. Gute Primärarztsysteme sind geprägt von guter (digitaler) Erreichbarkeit und klarer Orientierung. Die Patientensteuerung ist dort so gestaltet, dass sie von Patienten als bestmöglicher Zugang zu gesundheitlicher Versorgung empfunden wird. Verallgemeinern lässt sich dies anhand des verhaltensökonomischen Prinzips der Standardvoreinstellung (Default): Der koordinierte Versorgungspfad muss als naheliegende und vorteilhafte Standardoption gestaltet werden. Der unmittelbare Nutzen ist dabei zentral, da Patienten stärker auf kurzfristige Vorteile reagieren als auf abstrakte Systemziele [25].

Eine solche, das Prinzip des Nudgings nutzende Systemarchitektur ist in anderen Lebensbereichen bereits etablierte Praxis, in Form von Bonusprogrammen für bspw. Reisen oder Konsumgüter. So zeigen beispielsweise Vielfliegerprogramme von Airlines seit Jahren, wie individuelles Verhalten effektiv und akzeptiert gesteuert werden kann – ohne Zwang, aber mit klaren Anreizen. Warum sind dergleichen Programme interessant und auch auf das Gesundheitswesen übertragbar?

### Das Beispiel Vielfliegerprogramm und Übertragbarkeit

Bei einer Airline sitzen alle Passagiere – mit und ohne Vielfliegerstatus – in der gleichen Maschine und erhalten alle die gleiche Kernleistung in Form einer sicheren Beförderung ans gleiche Ziel. Qualitätsvorgaben für die Piloten und die Crew gelten für alle Passagiere, ohne 2‑Klassen-Gesellschaft. Das ist das Grundprinzip eines solidarischen Systems und so muss es auch im Gesundheitswesen sein. Weder beim Zugang zu Leistungen noch bei der Qualität der Behandlung darf es Unterschiede geben, genauso wenig wie zwischen privat und gesetzlich versicherten Patienten.

Im Flugverkehr wird gemäß Status geregelt, wer zuerst einsteigen darf und wer Zugang zur Lounge hat. Bei diesen Bonusprogrammen führt die Anzahl der Buchungen und damit das ausgegebene Geld zu einem höheren Status. Hier gibt es eine zentrale Abwandlung fürs Gesundheitswesen: Der Status hängt nicht von der Kaufkraft der Patienten ab, sondern von deren Verhalten und Compliance. Die Vorteile selbst sind wieder übertragbar: „Gesundheitsmeilen“, die man bspw. für Sportkurse oder eben für die Teilnahme an der Patientensteuerung erhalten hat, können für gewisse Vorteile eingelöst werden, z. B. Einzelbettzimmer oder Sachprämien.

Im Kontext der Übertragbarkeit ist aber entscheidend, dass dergleichen Bonusmodelle im Gesundheitswesen keine Unterschiede bei Behandlungsqualität oder Dringlichkeit erzeugen. Vorteile dürfen sich nicht auf eine bevorzugte medizinische Behandlung beziehen, sondern auf Service und Orientierung, etwa digitale oder telefonische Navigationsangebote, niedrigschwellige Terminorganisation nach medizinischer Dringlichkeit, reduzierte Zuzahlungen für präventionsbezogene Leistungen oder zusätzliche Gesundheitsberatung.

Sind diese Grundvoraussetzungen gegeben, kann ein gesundheitsbezogenes Bonusprogramm dazu beitragen, die koordinierte Inanspruchnahme von Leistungen zu fördern. Gleichzeitig können Versorgungspfade transparent gemacht werden und Prävention und Eigenverantwortung als feste Säulen der Gesundheitsversorgung können gestärkt werden. In Summe führt dies zu einer deutlich höheren Akzeptanz eines Primärarztsystems.

### Ein bundeseinheitliches Bonusprogramm für eine sozial ausgewogene Patientensteuerung

Ein Bonusprogramm sollte bundesweit einheitliche Leitplanken haben und könnte folgende Elemente umfassen:

(i) *Belohnung koordinierter Versorgung*, beispielsweise durch Punkte oder Boni für einen Erstkontakt über den Hausarzt oder eine vergleichbare Stelle mit Steuerungsfunktion;
(ii) *Belohnung präventiven Verhaltens*, beispielsweise durch Anreize für Vorsorgeuntersuchungen, Disease-Management-Programme oder evidenzbasierte Präventionsangebote; solche Vorteile gibt es bereits im zahnmedizinischen Bereich, der in Bezug auf Prävention und Eigenverantwortung durchaus Vorreiter ist [3];
(iii) *spürbare Vorteile für Versicherte*, beispielsweise in Form reduzierter oder erlassener Zuzahlungen oder digitaler Serviceleistungen, etwa eines bevorzugten Zugangs zu Online-Angeboten wie Ernährungsberatung oder Online-Sportkursen.

Gleichzeitig sind derlei Nudging-Ansätze nicht frei von sozialen Risiken. Sie können unbeabsichtigt jene Versicherten begünstigen, die ohnehin über höhere Gesundheitskompetenz, bessere digitale Zugänge und größere zeitliche Ressourcen verfügen. Gerade vulnerable Gruppen könnten Bonusvorteile schwerer erreichen, obwohl sie von koordinierter Versorgung besonders profitieren würden. Ein sozial ausgewogenes Modell muss daher barrierefrei, mehrsprachig und auch analog nutzbar sein und belohnt die Teilnahme an Versorgungsprozessen, keine gesundheitsbezogenen Ergebnisse. Bonusmodelle sollten deshalb mit Härtefallregelungen, niedrigschwelliger Beratung und einem kontinuierlichen Monitoring sozialer Verteilungswirkungen verbunden werden.

Um ein solches sozial ausgewogenes Modell umzusetzen, sind verschiedene politische Entscheidungen erforderlich. Der gesetzliche Rahmen für Bonusprogramme muss erweitert werden, die es den Krankenkassen ausdrücklich ermöglichen, verhaltensökonomisch fundierte Bonus- und Loyalitätsprogramme zur Patientensteuerung einzusetzen, über bisherige Präventionsboni hinaus. Der wesentliche Schritt, Patientensteuerung als Regelversorgung zu definieren, wurde in den Vorschlägen der Finanzkommission Gesundheit bereits berücksichtigt. Dem Gemeinsamen Bundesausschuss obliegt in diesem Zusammenhang, verbindliche Standards für digitale Ersteinschätzung, Navigation und koordinierte Versorgung sowie deren angemessene Vergütung festzulegen. Die Steuerung muss dabei multiprofessionell organisiert werden. Hausärzte können weiter die zentrale Rolle übernehmen, benötigen hierfür jedoch die koordinierte Unterstützung durch Pflegefachpersonen, medizinische Fachangestellte, Telemedizin und regionale Versorgungszentren. Für die Akzeptanz des Systems ist entscheidend, dass Patienten leicht nachvollziehen können, welche Versorgungswege für sie vorgesehen sind und welchen konkreten Nutzen diese bieten. Um dies zu erreichen, ist ein gutes Kommunikationskonzept unerlässlich [25].

## Fazit: Effizienz und Solidarität sind kein Widerspruch

Eine Transformation der GKV, die Patientensteuerung und eine zeitgemäße Definition von Eigenverantwortung integriert und beides für Patienten als die beste Wahloption gestaltet, stärkt nicht nur die Wirtschaftlichkeit, sondern auch die Solidarität. Ressourcen werden in solch einem System dort eingesetzt, wo sie medizinisch notwendig sind.

Ein Primärarztsystem als Teil eines anreizbasierten Steuerungssystems ist dann erfolgreich, wenn es für Patienten spürbare Vorteile wie kürze Wartezeiten oder eine verbesserte Versorgung bringt. Bonusprogramme nach dem Vorbild erfolgreicher Loyalitätsmodelle bieten die Chance, Patientensteuerung wirksam, akzeptiert und sozial ausgewogen zu gestalten. Patientensteuerung ist demnach keine Frage von Disziplin, sondern von Systemdesign. Die zentrale Transformationsfrage für das Gesundheitswesen lautet daher nicht: „Wie begrenzen wir Leistungen?“, sondern: „Wie gestalten wir ein System, das verantwortliches Verhalten ermöglicht, belohnt und strukturell unterstützt?“

Selbst moderate Steuerungseffekte können dabei bereits fiskalische Wirkungen entfalten.

Hinweise hierauf liefern beispielsweise Evaluationen der Hausarztzentrierten Versorgung. So zeigte sich in Baden-Württemberg, dass es bei Teilnehmenden der HZV im Jahr 2021 im Vergleich zur Kontrollgruppe zu 4,3 % geringeren Gesamtausgaben kam [26]. Der Effekt ergab sich insbesondere aus geringeren stationären Ausgaben und einer effizienteren Arzneimitteltherapie. Eine formale Hausarztbindung wie in der HZV reicht aber nicht aus, um Effizienzgewinne durch Patientensteuerung tatsächlich zu realisieren, sondern sie entstehen durch eine bessere Allokation knapper Versorgungskapazitäten. Patientensteuerung ist damit kein Instrument der Zugangsbeschränkung, sondern ihr wirtschaftlicher Nutzen entfaltet sich vor allem dann, wenn medizinisch nicht notwendige Kontakte und stationäre Aufenthalte vermieden, Doppeluntersuchungen reduziert, chronisch Kranke kontinuierlicher betreut und Arzneimitteltherapien sicherer gesteuert werden. Daraus folgende Effizienzgewinne entsprechen im Gesamtvolumen des GKV-Budgets schnell Milliardenbeträgen.

Die heutigen Finanzprobleme der GKV sind Ausdruck eines strukturellen Steuerungsdefizits. Patientensteuerung und Eigenverantwortung sind daher keine moralischen Appelle, sondern ökonomische Notwendigkeiten, um ein solidarisches System durch effiziente Organisation stabil zu halten.

## Data Availability

Alle dieser Arbeit zugrunde liegenden Daten sind in diesem Artikel enthalten.
